# K_2P_2.1 channels modulate the pH- and mechanosensitivity of pancreatic stellate cells

**DOI:** 10.1007/s00424-024-03021-z

**Published:** 2024-09-26

**Authors:** Micol Rugi, Verena Hofschröer, Zoltán Pethő, Benjamin Soret, Thorsten Loeck, Albrecht Schwab

**Affiliations:** 1 Institut Für Physiologie II, Robert-Koch-Str. 27B, 48149 Münster, Germany; 2 Laboratory of Cell Physiology, INSERM U 1003, Laboratory of Excellence Ion Channel Science and Therapeutics, Department of Biology, Faculty of Science and Technologies, University of Lille, 59650 Villeneuve d’Ascq, France

**Keywords:** K_2P_2.1 Channel, Pancreatic Stellate Cells, Mechanosensitivity, PH

## Abstract

Pancreatic stellate cells (PSCs) are central in the development of acute pancreatitis and tumor fibrosis in pancreatic ductal adenocarcinoma (PDAC). Fibrosis and a unique pH landscape represent characteristic properties of the PDAC microenvironment. Mechanosensitive ion channels are involved in the activation of PSCs. Among these channels, K_2P_2.1 has not yet been studied in PSCs. K_2P_2.1 channels are pH- and mechanosensitive. We confirmed K_2P_2.1 expression in PSCs by RT-qPCR and immunofluorescence. PSCs from K_2P_2.1^+/+^ and K_2P_2.1^−/−^ mice were studied under conditions mimicking properties of the PDAC microenvironment (acidic extracellular pH (pH_e_), ambient pressure elevated by + 100 mmHg). Migration and the cell area were taken as surrogates for PSC activation and evaluated with live cell imaging. pH_e_-dependent changes of the membrane potential of PSCs were investigated with DiBAC_4_(3), a voltage-sensitive fluorescent dye*.* We observed a correlation between morphological activation and progressive hyperpolarization of the cells in response to changes in pH_e_ and pressure. The effect was in part dependent on the expression of K_2P_2.1 channels because the membrane potential of K_2P_2.1^+/+^ PSCs was always more hyperpolarized than that of K_2P_2.1^−/−^ PSCs. Cell migration velocity of K_2P_2.1^+/+^ cells decreased upon pressure application when cells were kept in an acidic medium (pH_e_ 6.6). This was not the case in K_2P_2.1^−/−^ PSCs. Taken together, our study highlights the critical role of K_2P_2.1 channels in the combined sensing of environmental pressure and pH_e_ by PSCs and in coordinating cellular morphology with membrane potential dynamics. Thus, K_2P_2.1 channels are important mechano-sensors in murine PSCs.

## Introduction

Activated pancreatic stellate cells (PSCs) play an important role in several pancreatic pathologies such as acute and chronic pancreatitis [11] as well as in pancreatic ductal adenocarcinoma (PDAC) [10]. In PDAC, they produce large amounts of extracellular matrix so that they are in part responsible for fibrosis (desmoplasia) of the tissue in PDAC. Desmoplasia results in a rise of tissue pressure by up to 100 mmHg [27] which in turn maintains the activation of PSCs in PDAC [9, 28]. In addition, the PDAC microenvironment is characterized by a unique pH landscape. After each meal, pancreatic ductal cells secrete up to 150 mmol/l of HCO_3_^−^ into the ducts. This is inevitably accompanied by the equimolar secretion of H^+^ into the interstitial space, where PSCs reside [2, 22, 23, 33]. Hence, PSCs are adapted to cope with large variations of the extracellular pH (pH_e_). PSCs are highly responsive to pH_e_ changes, which are involved in their transition to the active state [24]. The intermittent stromal acidification of the healthy pancreas is one of the main factors that keeps PSCs in their quiescent state. In contrast, once PSCs have become activated after the onset of PDAC, the interstitial acidification of the PDAC stroma acts like a double-edged sword and further promotes their protumorous activity [24]. The question, however, remains by which mechanisms intra-/extracellular pH dynamics and mechanical properties of the PDAC microenvironment are sensed by PSCs and transduced into a distinct cell behavior.

We recently showed that ion channels of PSCs play important roles in sensing and transducing cues from the PDAC microenvironment [9, 15, 21, 28, 34]. These studies also highlighted that some ion channels, like Piezo1, may function as multi-modal sensors. Piezo1 is one of the main mechanosensitive ion channels of PSCs that regulates the migratory activity of these cells. It does so in a pH-dependent way [15] because an intra- or extracellular acidification blunts the activation of Piezo1. Thereby Piezo1 can integrate these two microenvironmental stimuli. Moreover, we revealed that TRPC1 channels, although most likely not being mechanosensitive themselves, regulate mechano-signaling of PSCs in response to elevations of the ambient pressure [9, 28].

Similarly, K_2P_2.1 channels (encoded by *KCNK2*; also described as TREK-1 (TWIK-related potassium channel-1)), which are also expressed in PSCs, are able to integrate multiple physical and chemical cues from the intra- and extracellular environment. They belong to the family of two pore-domain K^+^ channels which has 15 subunits and can be divided into six subfamilies [7]. K_2P_2.1 channels have a low and voltage-independent basal activity. In the context of pancreatic pathologies such as PDAC and for the present study, it is most notable that K_2P_2.1 channels exhibit both mechanosensitivity and pH sensitivity [29, 31]. The mechanosensitivity of K_2P_2.1 channels is viewed as a direct consequence of plasma membrane tension [17]. The crystal structure of K_2P_2.1 revealed critical domains that are highly affected by protonation [20]. pH modulates the functionality of K_2P_2.1 channels intracellularly [20] and extracellularly [6, 30]. Upon intracellular acidification, the opening of the channels occurs even in the absence of membrane stretch [16]. The opposite effect is observed when the acidification occurs extracellularly so that a decrease of pH_e_ leads to a progressive inhibition of the human K_2P_2.1 current. Notably, murine K_2P_2.1 have a lower sensitivity to pH_e_ (~ 35% inhibition) than the human variant [6]. In pancreatic cancer (BxPC-3) cells, the application of BL1249, an activator of K_2p_2.1, results in a hyperpolarization of the membrane potential comparable with the one elicited by shifting pH from pH_e_ 6.7 to pH_e_ 8.2. BL1249-induced currents showed a similar current-over-time signature as observed for the pH-sensitive current [30].

In the present study, we investigated how K_2P_2.1 channels impact the pH-dependent response of PSCs to an increased ambient pressure. We reasoned that K_2P_2.1 channels might counterbalance the depolarizing effect of Piezo1 and TRPC1 channels upon a mechanical stimulation (e.g., increased pressure). Our and others’ previous studies have shown that PSCs utilize these channels and also TRPV4 for sensing and/or transducing mechanical cues such as matrix stiffness and tissue pressure from the microenvironment [9, 15, 28, 35, 36]. We used cell migration and the cell area as readouts of PSC activation and combined them with measurements of the membrane potential.

## Materials and methods

### Isolation of murine primary pancreatic stellate cells (PSCs)

Animal experiments were conducted in accordance with the approval of the local animal welfare committee, permit no.: LANUV 81–02.05.50.20.003. Cells were isolated from 8- to 12-week-old male/female 129v/C57BL/6 J wild-type and *Kcnk2*^−/−^ mice (K_2P_2.1^−/−^), kindly provided by Prof. Sven Meuth, University of Düsseldorf, Germany [3, 12].

Primary PSCs were isolated from the pancreas as previously described [21, 24]. In short, the murine pancreas was digested enzymatically using 0.1% collagenase P (Roche Holding AG, Basel, Switzerland) at 37 °C for 30 min on an orbital shaker. The digested tissue was resuspended in GBSS buffer (Pan-Biotech GmbH, Aidenbach, Germany) and centrifuged (1040 g) at RT for 8 min. The supernatant was removed and the pellet was resuspended in cell culture medium: DMEM-F12 (SAFC, Taufkirchen, Germany) with 10% FCS superior (Sigma-Aldrich Chemie GmbH, Taufkirchen, Germany) and 1% penicillin/streptomycin (Pan-Biotech GmbH, Aidenbach, Germany), supplemented with 24 mM NaHCO_3_ to adjust for pH_e_ 7.4 or 4 mM NaHCO_3_ to adjust for pH_e_ 6.6 as calculated with the Henderson-Hasselbalch equation. Cells were seeded onto an FCS-coated dish for initial adhesion. Nonadherent cells were removed by forceful washing after 90 min. Freshly isolated PSCs were cultured in DMEM-F12 at pH_e_ 7.4, 37 °C, and 5% CO_2_ for 5 days after isolation. On day 6, cells were passaged and pH_e_ was changed to pH_e_ 7.4 or pH_e_ 6.6 for the following 3 days until the cells were harvested for experiments.

### Pressure application

Pressure was applied in custom-made pressure chambers (Feinmechanische Werkstätten, Medizinische Fakultät Münster; as described in [9, 19]). Cells were incubated at 100 mmHg above ambient atmospheric pressure. For cell migration experiments, pressure of 100 mmHg was applied for 24 h. For membrane potential measurements, cells were incubated in the presence of increased pressure (100 mmHg) for 24 h and 48 h before being analyzed.

### mRNA isolation and qRT-PCR analysis

RNA was isolated from wt (K_2P_2.1^+/+^) and K_2P_2.1^−/−^ PSCs in passage 2 using TRIzol™ (Invitrogen AG, Carlsbad, USA) and chloroform (Sigma-Aldrich Chemie GmbH), according to the manufacturer’s protocol [19, 24]. Resulting RNA concentrations were measured using a BioPhotometer (Eppendorf SE, Hamburg, Germany).

For cDNA synthesis, 2 µg RNA was reverse transcribed using SuperScript IV Reverse Transcriptase (Inivitrogen, Carlsbad, USA). After the reverse transcription, RT-qPCR was performed from 2 µl cDNA with a QuantStudio 3 thermal cycler (Thermo Fisher Scientific, Waltham, USA). The reaction mix contained 5 µl Syber Green (Thermo Fisher Scientific), 0.5 µl forward and 0.5 µl reverse primer (10 µM each), and 3 µl H_2_O. The following is the cycler protocol: initial DNA denaturation (95 °C, 2 min), 40 cycles of DNA denaturation (94 °C, 30 s), primer attachment (57 °C, 25 s), and DNA elongation (72 °C, 45 s) [19]. The evaluation of the data was performed according to the 2^−ΔCt^ method [18]. *Kcnk2* (K_2P_2.1) expression was normalized to the geometric mean of *Gapdh* and *Ywhaz* housekeeper gene expression. We used the following primers: K_2P_2.1 Forward 5′-ATACTGCAGGAGTGGCGG-3′ and K_2P_2.1 Reverse 5′-CAAGCACGGTGGGTTTTGAG-3′; GAPDH Forward 5′-GAAGGTCGGTGTGAACGGA-3′ and GAPDH Reverse 5′-GAAGATGGTGATGGGCTTCC-3′; YWHAZ Forward 5′-GATCCCCAATGCTTCGCAAC-3′ and YWHAZ Reverse 5′-TGACTGGTCCACAATTCCTTTCT-3′. The primers used to probe the expression of Piezo1, TRPM7, TRPV4, and TRPC1 in K_2P_2.1^+/+^-PSCs and K_2P_2.1^−/−^ PSCs were the same as described previously [9].

### Immunofluorescence staining

Glass bottom dishes (MatTeK Corporation, Ashland, USA) were coated with 0.001% poly-l-lysine (Sigma-Aldrich Chemie GmbH) at RT for 20 min and washed twice with PBS. A total of 25,000 PSCs per dish were seeded and incubated at 37 °C and 5% CO_2_ to adhere overnight. The cells were fixed with 4% paraformaldehyde (PFA; Carl Roth GmbH & Co. KG, Karlsruhe, Germany) at 4 °C for 30 min. Samples were washed three times with PBS. For permeabilization and blocking, cells were treated with 0.1% saponin (Sigma-Aldrich)/10% FCS diluted in PBS at RT for 1 h, and then washed twice using PBS. Samples were incubated with a rabbit polyclonal anti-K_2P_2.1 antibody (# APC-047, Alomone Labs, Israel), diluted 1:100 in PBS containing 0.1% saponin/1% FCS at 4 °C overnight. After washing, the cells were incubated with the secondary antibody alexa-488 goat anti-rabbit (1:1000, # A11034, Invitrogen Thermo Fisher Scientific) in the dark at RT for 30 min. Cells were washed again and DAPI (1:10,000; Sigma-Aldrich Chemie GmbH) was added. Immunofluorescence images were acquired with a Zeiss Axiovert 200 inverted fluorescence microscope (Zeiss, Oberkochen, Germany) at 40 × or 100 × magnification.

### Membrane potential measurements

We measured the membrane potential of freshly isolated PSCs that were cultured for up to 48 h with the fluorescent voltage-sensitive dye DiBAC_4_(3) (Bis-(1,3-dibutylbarbituric acid) trimethine oxonol; AAT Bioquest, Pleasanton, USA). When the membrane potential depolarizes, the anionic DiBAC_4_(3) enters the cell so the fluorescent signal gets brighter [1]. Conversely, DiBAC_4_(3) leaves the cytosol during plasma membrane hyperpolarization so that fluorescence intensity decreases.

Freshly isolated PSCs were seeded on glass bottom dishes coated with a diluted (1:10) collagen-based extracellular matrix, polymerized overnight at 37 °C. This matrix consists of 10.4 g/l RPMI (Sigma-Aldrich), 10 mmol/l HEPES (Sigma-Aldrich), 40 µg/ml laminin (Sigma-Aldrich), 40 µg/ml fibronectin (Corning B.V. Life Sciences, Amsterdam, Netherlands), 5.4 µg/ml collagen IV (Corning B.V. Life Sciences), 12 µg/ml human collagen III (Corning B.V. Life Sciences), and 500 µg/ml collagen I (Biochrom GmbH, Berlin, Germany); pH was adjusted to pH 7.4 with NaOH. Pressure and/or pH_e_ conditions were applied for up to 48 h as indicated. Prior to the experiments, cells were washed twice with PBS and incubated with HEPES-buffered medium at 37 °C for 2 h to equilibrate the intracellular pH. After 2 h, cells were incubated with 2 µM DiBAC_4_(3) in 0.1% DMSO for 20 min. We used Ringer’s solution (37 °C) with the following composition (in mmol/L): 140 NaCl, 5.4 KCl, 1.2 CaCl_2_, 0.8 MgCl_2_, 5.5 glucose, and 10 HEPES, titrated to pH_e_ 7.4 or pH_e_ 6.6 with 1 M NaOH and complemented with 2 µM DiBAC_4_(3).

The imaging setup consisted of a fluorescence microscope (Zeiss Axiovert 100, Zeiss, Oberkochen, Germany) with a 40 × oil objective, polychromator generating an excitation wavelength of 490 nm, high-speed shutter, beam splitter 515 dcxr and D535/25 m emission filter, and a sCMOS pco.edge camera (Visitron Systems GmbH, Puchheim, Germany). Images were acquired every other 10 s. Image acquisition was controlled by VisiView software (Visitron Systems).

**Fig. 1:**
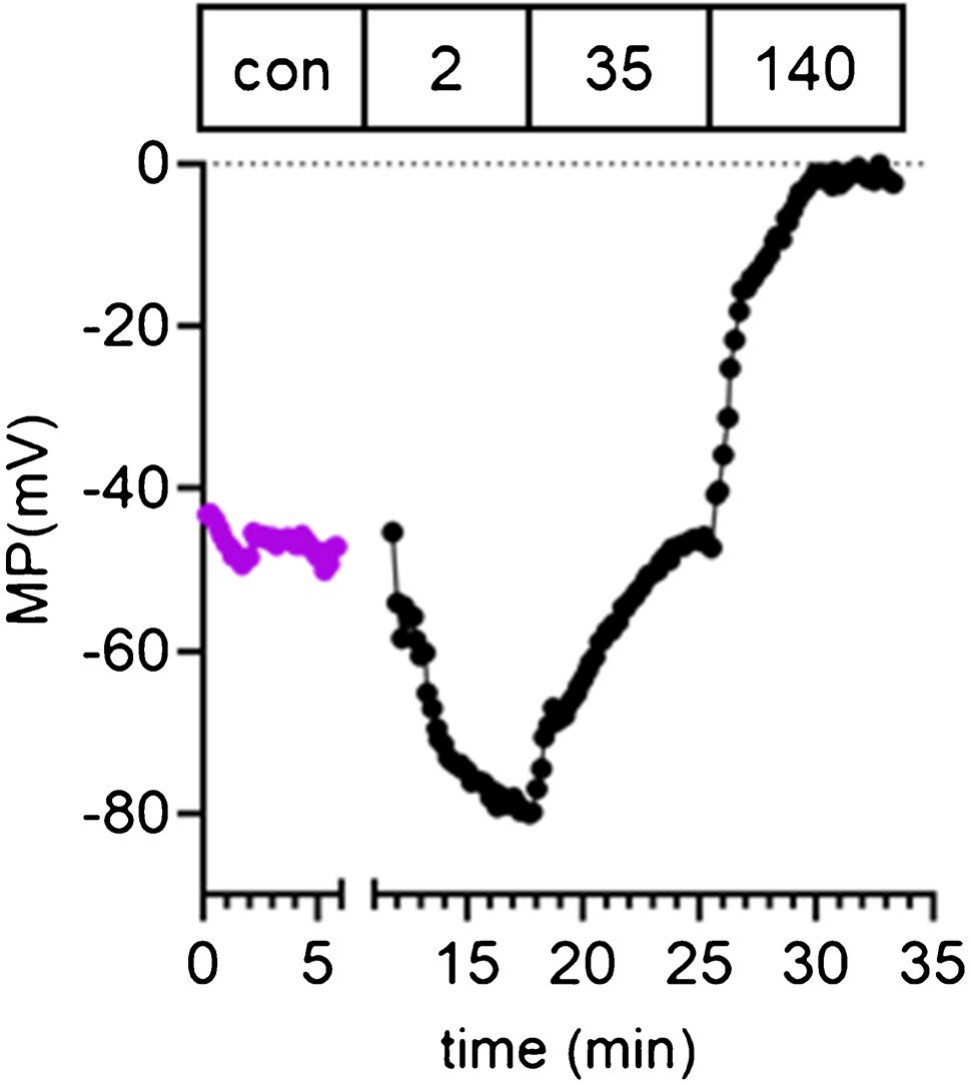
Original tracing of a membrane potential measurement of a K_2P_2.1^+/+^ PSC kept at pH_e_ 7.4. The control period (con) is shown in purple. The Na^+^ concentration (in mM) of the calibration solutions is indicated

The cells were continuously superfused with DiBAC_4_(3)-containing solution during the entire course of the experiment. Figure 1 displays an original tracing of a membrane potential recording of a K_2P_2.1^−/−^ PSC. First, cells were superfused with Ringer’s solution corresponding to their culture pH for several minutes. Next, the calibration was performed with three different glucose-free Ringer’s solution containing 2 mM Na^+^, 35 mM Na^+^, and 140 mM Na^+^ (NaCl was iso-osmotically replaced by NMDG-Cl). Additionally, 1 µM of the ionophore gramicidin was added to each of the calibration solutions (diluted in UVAsol; Merck KGaA, Darmstadt).

The fluorescence intensity values obtained during the calibration allowed to calculate the plasma membrane potential of PSCs by using the Goldman-Hodgkin-Katz equation as previously described [19]. Data were analyzed using NIH ImageJ software and the intensity values were background corrected. The minimum correlation factor (*R*^2^) of the linear regression lines of the calibration was set to 0.9. Values below this cutoff were excluded from further analysis.

### Cell migration experiments

A total of 25,000 cells were seeded in a flask pre-coated with a thin layer of extracellular matrix as described for the membrane potential measurements and incubated overnight. Migration of K_2P_2.1^+/+^ and K_2P_2.1^−/−^ PSCs was recorded under control conditions (normal ambient pressure), after being exposed to 100 mmHg above atmospheric pressure at 37 °C and 5% CO_2_ for 24 h and then maintained at normal ambient pressure. Alternatively, we recorded migration in the presence of an acutely elevated ambient pressure (+ 100 mmHg).

Single PSC migration was observed with time-lapse video microscopy using an inverted microscope (Axiovert 40C, Carl Zeiss Inc.) at 37 °C as previously described [19, 24, 32]. Images were acquired in 5-min intervals for 6 h. Image stacks were segmented using the Amira® 2019 software (Thermo Fisher Scientific, Waltham, USA). Velocity (µm/min), translocation (µm), and the projected cell area (µm^2^) were quantified. A maximum of ten cells were randomly analyzed per each image stack. The velocity was calculated by applying a three-point difference quotient. Translocation was defined as the net distance between the positions of the PSCs at the start and the end of the experiment.

### Statistical analysis

Statistical analyses were performed with GraphPad Prism 9 software (GraphPad Software, Inc.). All data are shown as mean ± SEM. Each experiment was replicated independently with cells from three to four different animals; *N*/*n* = number of animals/number of analyzed cells. The normal distribution of the data was tested with Shapiro–Wilk test. Unpaired Student’s *t*-test was used when two groups were compared. In case of nonparametric distribution, the Mann–Whitney *U* test was applied. Experiments involving more than two groups were analyzed using one-way ANOVA and Tukey’s multiple comparisons test for normally distributed data. The Kruskal–Wallis test and Dunn’s multiple comparisons test were used for nonparametric data. For all tests, *p*-values < 0.05 were considered statistically significant.

## Results

### K_2p_2.1 is expressed in primary PSCs

**Fig. 2:**
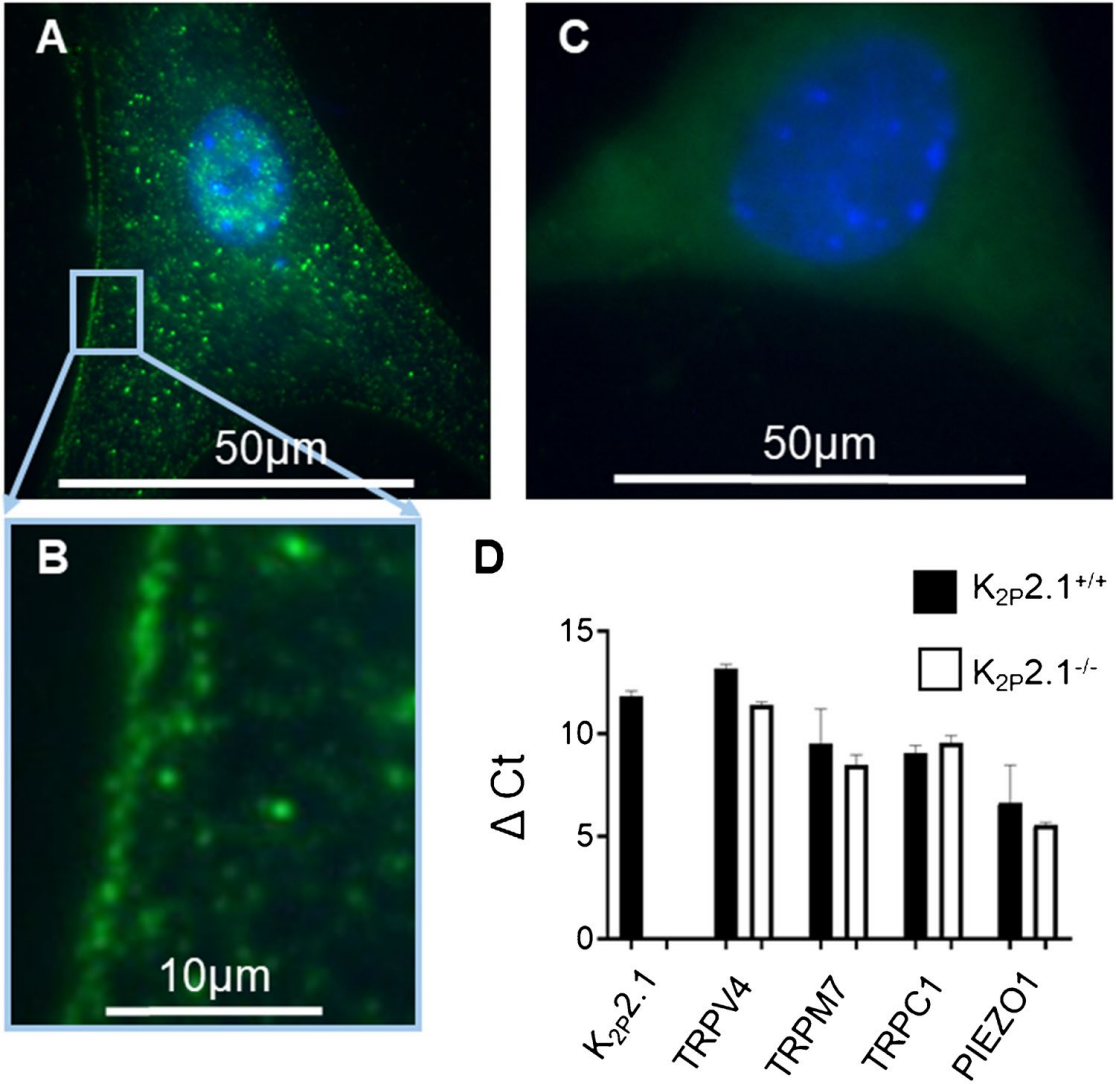
Expression of mechanosensitive ion channels in PSCs. **A** Immunostaining of K_2P_2.1 channels (green) in the plasma membrane of PSCs. The DAPI-stained nucleus is labeled in blue. **B** Zoom-in to show the membrane localization of K_2P_2.1 channels in the plasma membrane of PSCs. **C** Control experiments in the absence of the primary antibody. The DAPI-stained nucleus is labeled in blue.** D** The mRNA expression of TRPV4, TRPM7, TRPC1, and PIEZO1 channels were not affected by the knockout of K_2P_2.1 channels (K_2P_2.1.^−/−^). Expression levels are shown relative to mRNA expression of the housekeeping genes GAPDH and YWHAZ (*N* = 3, *n* ≥ 30)

We had shown previously by means of RT-qPCR that K_2P_2.1 channels are expressed in PSCs [9]. To confirm the presence of K_2P_2.1 in the plasma membrane, we performed immunofluorescence staining (Fig. 2A–C). Images depicted the typical “dots pattern” which is usually observed for ion channel staining. RT-qPCR (Fig. 2D) confirmed the absence of K_2P_2.1 mRNA in K_2P_2.1^−/−^ cells which is in line with [3]. Other mechanosensitive ion channels (TRPV4, TRPM7, TRPC1, and Piezo1) were expressed at similar levels in both K_2P_2.1^+/+^ and K_2P_2.1^−/−^ PSCs. Piezo1, as expected and published in [9], was the most highly expressed mechanosensitive ion channel.

### The membrane potential of PSCs is pH_e_-dependent

**Fig. 3:**
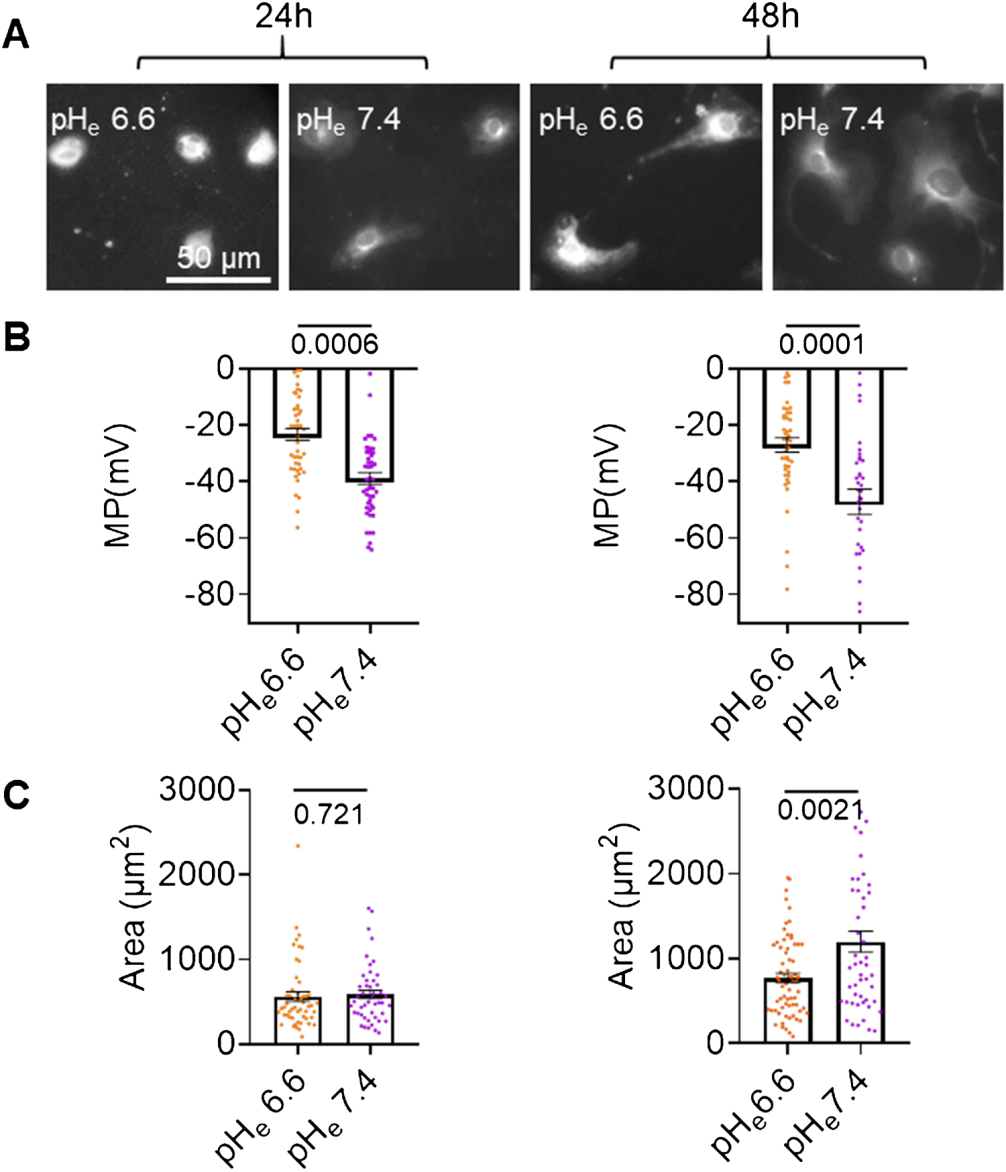
Membrane potential and cell size of PSCs depend on pH_e_. **A** Micrographs of PSCs loaded with the fluorescent voltage sensor DiBAC_4_(3). **B** Summary of membrane potential measurements: The membrane potential of K_2P_2.1.^+/+^ PSCs was more hyperpolarized when they were cultured at pH_e_ 7.4 than at pH_e_ 6.6. This effect was more pronounced after 48 h of culture. **C** PSCs cultured at pH_e_ 7.4 increased their cell area within 48 h, while this was not the case at pH_e_ 6.6. *N* = 3, *n* ≥ 30

At present, there is only limited information pertaining to the membrane potential of primary PSCs (firstly described in [19]). Here, we studied freshly isolated murine PSCs 24 h and 48 h post-isolation. In our previous study, we used PSCs in passage 2 that had been in culture for ~ 10 days [19]. Since PSCs are physiologically exposed to intermittent episodes of marked extracellular acidity [23], we initially maintained freshly isolated PSCs in an acidic environment (pH_e_ 6.6). Alternatively, freshly isolated PSCs were cultured at pH_e_ 7.4. Thereby, we identified alterations of the membrane potential (Fig. 3A and B) that occurred as a consequence of the transition from the quiescent immunomodulatory to the activated myofibroblastic phenotype of PSCs [24]. PSCs had a depolarized membrane potential that remained essentially constant when kept at pH_e_ 6.6 for 24 h and 48 h, respectively (− 23.3 ± 2.1 mV and − 27.0 ± 2.5 mV; *p* = 0.892). In contrast, the membrane potential of PSCs kept at pH_e_ 7.4 was already more hyperpolarized at *t* = 24 h (− 38.0 ± 2.1 mV). There was a trend towards further hyperpolarization at *t* = 48 h: − 47.1 ± 4.5 mV; *p* = 0.203. Membrane potential dynamics were accompanied by changes of the morphology of PSCs (Fig. 3C). The projected area of PSCs did not change over time and remained small until *t* = 48 h when they were cultured at pH_e_ 6.6. In contrast, the size of PSCs increased after 24 h and 48 h at pH_e_ 7.4. Since the biggest differences of membrane potential and morphology were observed after 48 h, this time interval was chosen for the next set of experiments.

### The loss of K_2P_2.1 channels modulates the morphology and the membrane potential of PSCs

**Fig. 4:**
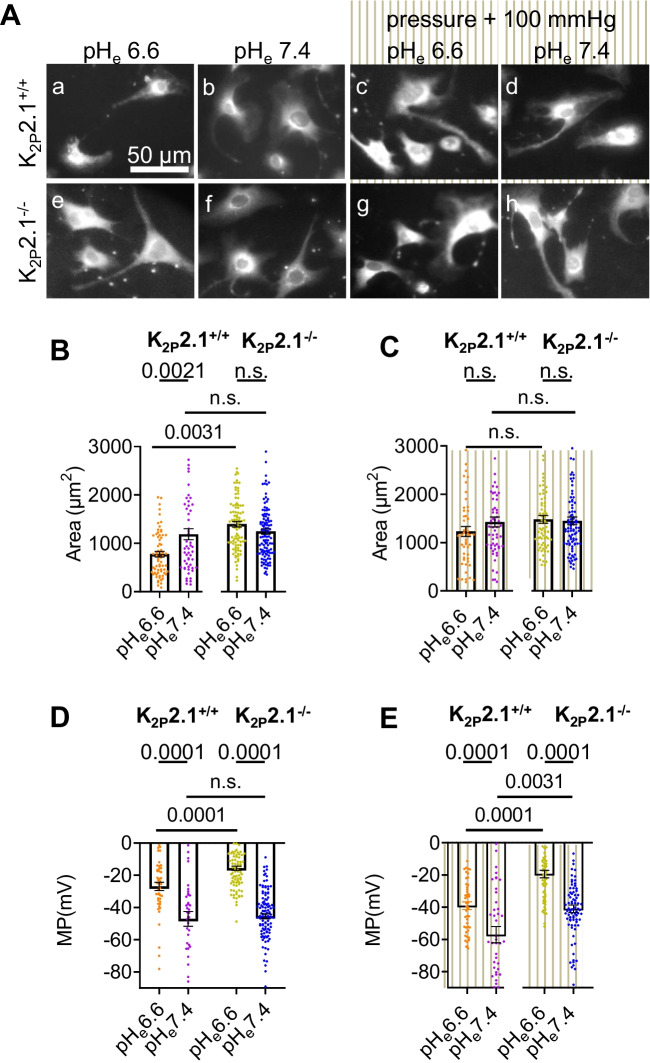
The morphology and membrane potential of PSCs are regulated K_2P_2.1-dependently when challenged by altered pH_e_ and ambient pressure. **A** K_2P_2.1^+/+^ and K_2P_2.1^−/−^. PSCs were cultured at the indicated pH_e_ at ambient pressure for 48 h. Grey stripes indicate culture conditions in the presence of an elevated ambient pressure (+ 100 mmHg) (c, d, g, h). Cells were loaded with the fluorescent voltage-sensitive dye DiBAC_4_(3). (a) K_2P_2.1^+/+^ PSCs cultured at pH_e_ 6.6 were small, consistent with their inactivated state [24]. (b) The size of K_2P_2.1^+/+^ PSCs increased when they were cultured at pH_e_ 7.4. (c, d) Culturing K_2P_2.1^−/−^ PSCs at ambient or elevated (+ 100 mmHg) pressure for 48 h resulted in an increased size independently of pH_e_. (e, f) K_2p_2.1.^−/−^ PSCs had an increased size regardless of the ambient conditions. They responded neither to changes of pH_e_ (e, f) nor to the application of pressure (g, h). **B** and **C** Summary of the morphometric analyses of PSCs cultured at ambient pressure (**B**) or at an elevated pressure (**C**). (*N* ≥ 3, *n* ≥ 30). **D** and **E** Summary of the membrane potential measurements under ambient pressure (**D**) and under elevated pressure (**E**). Application of pressure caused the membrane potential of PSCs to hyperpolarize in a partially K_2P_2.1-dependent way (*N* ≥ 3, *n* ≥ 30)

To investigate the impact of K_2P_2.1 channels on the membrane potential, we exposed freshly isolated K_2P_2.1^+/+^ and K_2P_2.1^−/−^ PSCs to an increased ambient pressure (+ 100 mmHg) at pH_e_ 6.6 and pH_e_ 7.4 for 48 h. The results are shown in Fig. 4.

The mechanosensitivity of PSCs depends at least in part on the expression of K_2P_2.1 channels. K_2P_2.1 channels affected the morphological changes of PSCs in response to pH_e_ and pressure (highlighted by grey stripes) in a distinct way. The projected area of K_2P_2.1^+/+^ PSCs was smaller at pH_e_ 6.6 than at pH_e_ 7.4: (773.1 ± 56.6 μm^2^ versus 1198.1 ± 122.7 μm^2^; see Figs. 3C and 4B). Elevating the ambient pressure by 100 mmHg significantly increased the cell area of K_2P_2.1^+/+^ PSCs at pH_e_ 6.6 (1249.0 ± 105.8 μm^2^; *p* = 0.0021) but not at pH_e_ 7.4 (1426.0 ± 102.9 μm^2^; *p* = 0.9997; Fig. 4B). On the contrary, the cell area of K_2p_2.1^−/−^ PSCs was not affected by any of our maneuvers (Fig. 3B and C). It amounted to 1393.5 ± 57.7 μm^2^ at pH_e_ 6.6 and to 1243.0 ± 57.4 μm^2^ at pH_e_ 7.4. Application of pressure did not change the size of K_2P_2.1^−/−^ PSCs any further. Pressure at pH_e_ 6.6: 1480.8 ± 79.9 μm^2^ and pressure at pH_e_ 7.4: 1448.8 ± 79.3 μm^2^.

The exposure to pressure shifted the membrane potential of K_2P_2.1^+/+^ PSCs kept at pH_e_ 6.6 from a depolarized value (− 27.0 ± 2.5 mV; as shown in Fig. 3) to more hyperpolarized values (− 39.0 ± 1.3 mV, Fig. 4D and E). Such a shift was also observed when K_2P_2.1^+/+^ PSCs were cultured at pH_e_ 7.4. K_2P_2.1^+/+^ PSCs reacted to the mechanical stimulation of pressure by further hyperpolarizing their membrane potential to − 57.0 ± 5.0 mV (Fig. 4E). The membrane potential of K_2P_2.1^−/−^ PSCs was even more depolarized at pH_e_ 6.6 than that of K_2P_2.1^+/+^ PSCs: − 15.6 ± 2.4 mV versus − 27.0 ± 2.52 mV. Notably, the membrane potential of K_2P_2.1^−/−^ PSCs did not hyperpolarize when the cells were exposed to pressure at pH_e_ 6.6 (− 19.4 ± 2.26 mV; Fig. 4D and E). The response of K_2P_2.1^−/−^ PSCs to mechanical stimulation was also altered at pH_e_7.4. The membrane potential of K_2P_2.1^−/−^ PSCs was more depolarized after the pressure application (− 40.9 ± 2.3 mV) than under control conditions (− 45.5 ± 1.5 mV) which is consistent with the activation of other mechanosensitive non-selective cation channels such as Piezo1.

### K_2P_2.1 controls the pH-dependent pressure sensitivity of migrating PSCs

**Fig. 5:**
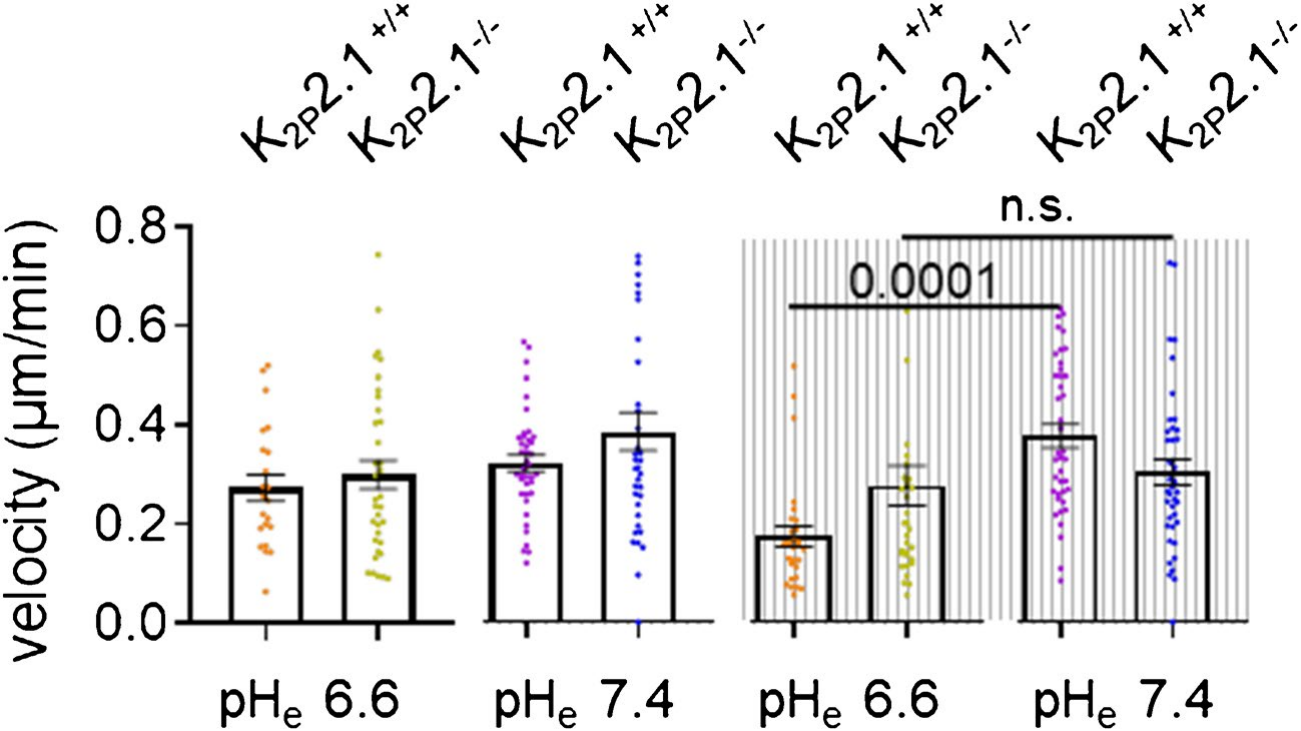
K_2P_2.1 channels allow migrating PSCs to respond to a simultaneous change of pH_e_ and ambient pressure. Migration of K_2P_2.1^+/+^ PSCs is regulated K_2P_2.1-dependently by the combination of pH_e_ and ambient pressure. Migration of K_2P_2.1^+/+^ PSCs is slowed down when the ambient pressure is increased (+ 100 mmHg) in the presence of an acidic medium (pH_e_ 6.6). K_2p_2.1.^−/−^ PSCs failed to react to this combined treatment and the velocity remained unchanged (*N* ≥ 3, *n* ≥ 30)

In the first set of migration experiments, we tested whether the presence or absence of K_2P_2.1 affected the response to pH_e_ (Fig. 5). There was only a trend for K_2P_2.1^−/−^ PSCs to move faster and further than K_2P_2.1^+/+^ PSCs: 0.38 ± 0.03 µm/min versus 0.32 ± 0.01 µm/min.

However, the combined application of pressure and an acidic pH_e_ disclosed differences between K_2P_2.1^+/+^ and K_2P_2.1^−/−^ PSCs (Fig. 5). PSCs were incubated in the presence of an elevated pressure (+ 100 mmHg) at the indicated pH_e_ for 24 h. Migration was then recorded under normal ambient pressure, i.e., during the recovery from a pressure load. In K_2P_2.1^+/+^ PSCs, pressure slightly increased the speed of the majority of the cells at pH_e_ 7.4 (+ 100 mmHg: 0.37 ± 0.02 µm/min versus control: 0.32 ± 0.01 µm/min), as already shown by [9]. Notably, pressure had the opposite effect at pH_e_ 6.6. K_2P_2.1^+/+^ PSCs slowed down when pressure was applied at pH_e_ 6.6 (+ 100 mmHg: 0.17 ± 0.02 µm/min versus control: 0.27 ± 0.02 µm/min). In contrast, K_2P_2.1^−/−^ PSCs failed to react to the pressure stimulation. Velocity remained unchanged regardless of pH_e_ (pH_e_ 7.4 + 100 mmHg: 0.30 ± 0.02 µm/min versus control 0.38 ± 0.03 µm/min; pH_e_ 6.6 + 100 mmHg: 0.27 ± 0.04 µm/min versus control 0.30 ± 0.02 µm/min). These results highlight the impact of K_2P_2.1 channels for pressure sensing of PSCs in the acidic PDAC microenvironment.

### K_2P_2.1 regulates PSC migration pH-dependently in response to acute pressure stimulation

**Fig. 6:**
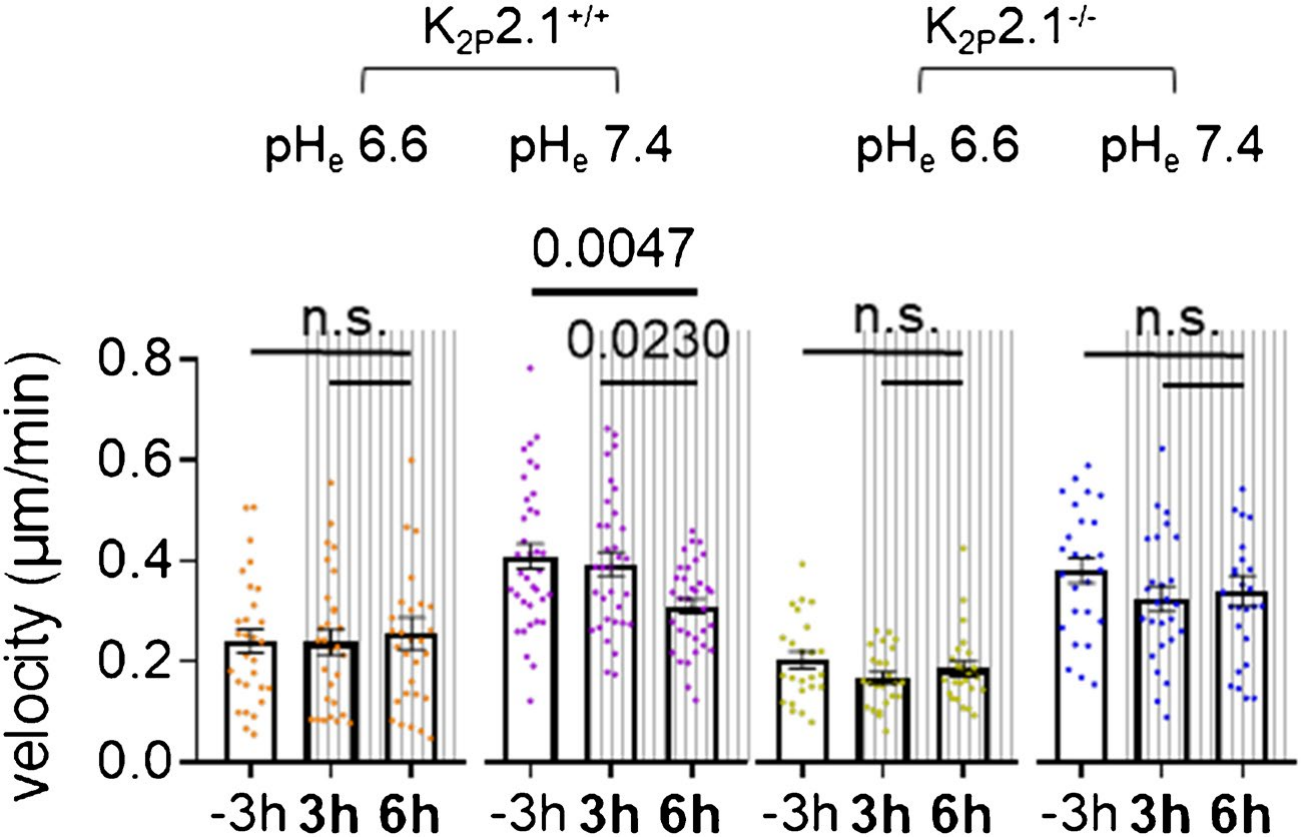
K_2P_2.1 channels confer sensitivity of PSCs to acute changes of ambient pressure (+ 100 mmHg). Migration of PSCs was recorded at pH_e_ 6.6 or pH_e_ 7.4 for 3 h prior to rising the ambient pressure by 100 mmHg (− 3 h) and then for the ensuing 6 h in the presence of the elevated pressure (+ 3 h, + 6 h). Average velocities were calculated for 3-h intervals (*N* ≥ 3, *n* ≥ 30). The main differences were detected at pH_e_ 7.4. K_2P_2.1^+/+^ PSCs decreased their velocity upon the exposure to pressure. K_2p_2.1.^−/−^ PSCs did not show this reduction, pointing to a loss of their pressure sensitivity (*N* ≥ 3, *n* ≥ 30)

Next, we analyzed the migratory behavior of PSCs following an acute pressure stimulus. For the first 3 h, PSCs migrated under normal atmospheric pressure. Then, pressure was increased by 100 mmHg and migration was recorded for another 6 h (binned in 3-h intervals). The results are shown in Fig. 6. An acidic pH_e_ 6.6 reduced the velocity for both cell types and overrode any potential effect of pressure (K_2P_2.1^+/+^: − 3 h (control): 0.24 ± 0.02 µm/min; + 3 h: 0.23 ± 0.02 µm/min; + 6 h: 0.25 ± 0.03 µm/min; K_2P_2.1^−/−^: − 3 h (control): 0.20 ± 0.01 µm/min; + 3 h: 0.16 ± 0.01 µm/min; + 6 h 0.18 ± 0.01 µm/min). When PSCs are kept at pH_e_ 7.4, the acute application of pressure also discloses the relevance of K_2P_2.1 channels in pressure sensing. Under control conditions (Fig. 6; − 3 h), the velocity of K_2P_2.1^+/+^ PCSs is 0.40 ± 0.023 µm/min and remained constant during the first 3 h of pressure treatment: 0.38 ± 0.023 µm/min (pressure on + 3 h). After + 6 h, however, the velocity was reduced to 0.30 ± 0.001 µm/min. In contrast, the K_2P_2.1^−/−^ PSCs lacked this response. Their migration velocity was unchanged after application of pressure (− 3 h: 0.38 ± 0.02 µm/min; + 3 h: 0.32 ± 0.02 µm/min; + 6 h: 0.34 ± 0.03 µm/min).

## Discussion

PDAC is characterized by a complex microenvironment. Its mechanical properties such as the elevated tissue pressure and the unique pH landscape have a marked impact on the function and differentiation of PSCs [9, 24–26]. This study sheds light on the functional relevance of K_2P_2.1 channels in regulating the membrane potential of PSCs under pathophysiologically relevant pH_e_ and pressure conditions. We believe that our findings reveal significant insights into PSC activation and mechanisms of migration occurring in the PDAC microenvironment.

Previous knowledge about the membrane potential of fibroblasts and even more so of PSCs is very limited. We found that the membrane potential of primary PSCs is around − 47 mV when kept under control conditions at pH_e_ 7.4. This aligns well with our previous findings where we reported a membrane potential of ~  − 40 mV in murine PSCs that had been in culture for ~ 10 days [19]. In hepatic stellate cells, a resting (zero current) membrane potential of − 81 mV was reported [14]. However, it has to be noted that this value is derived from whole-cell patch clamp experiments employing solutions with an unphysiological composition. Another patch clamp study performed on rat atrial fibroblasts described a zero current membrane potential of − 37 mV. Stimulating these cells by poking them with a micropipette induced a depolarization to − 10 mV while stretch caused a hyperpolarization from − 30 to − 45 mV [13]. Thus, the reported values and also our own results point to a large variability of the membrane potential of stellate cells or fibroblasts. In our view, likely explanations for this apparent scatter are methodological reasons such as the composition of the respective experimental solutions used for the patch clamp recordings. Moreover, the state of differentiation/activation of the cells has to be taken into account.

Our results reveal that K_2P_2.1 channels regulate the membrane potential of murine PSCs. The absence of K_2P_2.1 channels leads to a persistent depolarization of the membrane potential, regardless of the microenvironmental stimulation. The depolarization is even more pronounced when PSCs are cultured at pH_e_6.6. We interpret our measurements of the cell membrane potential as consistent with the intracellular rather than the extracellular acidification being the dominant regulator of the channel in PSCs. We know from our previous study that pH_i_ of PSCs follows pH_e_ when the latter is altered for a prolonged period of time [24]. This interpretation is supported by preliminary experiments in which we saw hardly any change of the membrane potential when the extracellular pH was changed for a period of only 3 min. In our view, the dual pH-dependent regulation from the intra- and extracellular sides allows to maintain a basal activity of K_2P_2.1 channels in the acidic PDAC microenvironment. Even then, K_2P_2.1 channels can function as background leak channels and stabilize the negative membrane potential.

The altered membrane potential of K_2P_2.1^−/−^ PSCs, in turn, is accompanied by altered migration and cell size. PSCs are known to utilize ion channels and transporters as sensitive tools to probe and respond to constituents of the microenvironment. Examples include K_Ca_3.1, Piezo1, TRPC1, TRPV4, and NHE1 [4, 9, 15, 21, 24, 28, 36]. Here, we add K_2P_2.1 channels to this list of transport proteins. We show that the pH- and mechanosensitivity of K_2P_2.1 channels make them ideal sensors of the acidic and pressurized PDAC microenvironment. K_2P_2.1 channels are clearly functional in acidic pH_e_ although with a reduced activity [6]. In our study, this is reflected by the observation that the membrane potential of K_2P_2.1^+/+^ PSCs is more hyperpolarized at pH_e_ 6.6 than that of their K_2P_2.1^−/−^ counterparts. Thus, K_2P_2.1 channels are at least partially responsible for maintaining a negative membrane potential in an acidic environment, where many other channels are less active (reviewed by [26]). Thereby, they can maintain the electrical driving force for other mechanosensitive channels such as Piezo1 and TRPV4.

We linked the K_2P_2.1-dependent membrane potential dynamics at pH_e_ 6.6 to a smaller projected area and to slower cell migration. Indeed, there is a link between plasma membrane depolarization and inhibition of rat myofibroblast proliferation, as well as an increase in contractility, while hyperpolarization promotes proliferation [5]. Moreover, we previously described a link between acidic pH_e_ and an immunomodulatory phenotype of murine PSC phenotype, while a myofibroblastic phenotype is linked to pH_e_ 7.4 [24]. Our results indicate that the membrane potential of PSCs gradually hyperpolarizes during the first 48 h after isolation. Interestingly, isolated PSCs gradually acquire a myofibroblastic phenotype within the first 72 h after isolation [24]. So far, it is still speculative whether the pH_e_-induced and K_2P_2.1-dependent membrane potential dynamics are part of the signaling cascade underlying the switch between immunomodulatory and myofibroblastic PSC phenotypes. Our findings are consistent with the idea that tightly regulated membrane potential dynamics in PSCs may serve as a protective mechanism against uncontrolled cell differentiation towards a myofibroblastic phenotype. Accordingly, disturbed membrane potential dynamics in K_2P_2.1^−/−^ PSCs are accompanied by an early increase of cell area after isolation, which is an indicator of the myofibroblastic differentiation. Under acidic conditions, K_2P_2.1 would thus reduce the likelihood of activation, i.e., myofibroblastic differentiation. Once pH_e_ rises to pH7.4, PSCs become more hyperpolarized promoting their transformation into myofibroblasts. It is conceivable that membrane potential dynamics are instrumental for Ca^2+^ signaling which is known to play a role in PSC activation [37]. Clearly more experiments are needed in support of our idea that K_2P_2.1 channels are modulating the pressure- and pH_e_-dependent differentiation of PSCs.

Previously, an inverse relationship has been observed between channel activity and morphogenic effects, indicating that the presence of K_2P_2.1 channels, rather than their function, influences cell morphology [16]. Additionally, the absence of K_2P_2.1 channels impairs actin cytoskeleton assembly in primary mouse brain microvascular endothelial cells, leading to a specific reduction in F-actin content within K_2P_2.1^−/−^ cells [3]. At first sight, our findings that pressure and pH_e_ regulate PSC migration and morphology of PSCs K_2P_2.1 dependently appear to be consistent with the known connection of K_2P_2.1 channels with the cytoskeleton. However, our observation that the projected area of K_2P_2.1^−/−^ PSCs is almost twice as large as that of the wild-type counterparts cannot be explained with a direct channel-cytoskeleton interaction. It rather argues for additional mechanisms such as a lack of mechano-sensing or mechano-signaling. It is reminiscent of a finding we made earlier with siTRPC1 cells. The defect of mechano-signaling in siTRPC1 cells was accompanied by a doubling of cell area and a seemingly unbalanced, high protrusive activity of lamellipodia [8]. Future studies are clearly warranted to investigate the molecular interactions between K_2P_2.1 channels and the cytoskeleton in greater detail.

## Data Availability

No datasets were generated or analysed during the current study.
